# CRISPR-Cas9-directed gene therapy for spinocerebellar ataxia type 1

**DOI:** 10.1016/j.omtn.2024.102377

**Published:** 2024-11-14

**Authors:** Mihai Miclăuș, Gabriel Balmus

**Affiliations:** 1 UK Dementia Research Institute at the University of Cambridge, Cambridge CB2 0AH, UK; 2 Department of Clinical Neurosciences, University of Cambridge, Cambridge Biomedical Campus, Cambridge CB2 0AH, UK; 3 Department of Molecular Neuroscience, Transylvanian Institute of Neuroscience, 400191 Cluj-Napoca, Romania

## Main text

Spinocerebellar ataxia type 1 (SCA1) is a debilitating neurodegenerative disorder characterized by progressive motor dysfunction and cerebellar degeneration resulting from a CAG trinucleotide repeat expansion in the ATXN1 gene. Despite extensive research efforts, effective treatments remain elusive. Current interventions are limited to symptomatic management or transient knockdown of ATXN1 expression using antisense oligonucleotides and RNA interference, approaches that require repeated administration and rely on endogenous cellular mechanisms.^1^ The advent of CRISPR-Cas9 (clustered regularly interspaced short palindromic repeats associated with CRISPR-associated protein 9) gene editing technology offers a transformative approach by enabling precise, long-lasting genomic modifications.^2^

In this issue of *Molecular Therapy Nucleic Acids*,^3^ Kelly J. Fagan and colleagues from the Beverly L. Davidson laboratory used CRISPR-Cas9 gene editing to target and reduce ATXN1 expression. Employing this strategy, they achieved a significant knockdown of ATXN1 mRNA and protein levels in human cells and a mouse model of SCA1, which correlated with improved motor coordination. This work underscores the versatility and efficacy of CRISPR-Cas9, which offers a long-term therapeutic solution for SCA1.

Like Huntington’s disease and other spinocerebellar ataxias, SCA1 is a repeat expansion disorder characterized by pathological expansions of trinucleotide DNA repeats. In SCA1, the expansion of CAG repeats in exon 8 of the ATXN1 gene leads to a mutant ATXN1 protein with an extended polyglutamine (polyQ) tract. This mutant protein accumulates predominantly in cerebellar Purkinje cells, causing a toxic gain of function wherein the polyQ-ATXN1 exerts deleterious effects through interactions with its native partner, capicua (CIC). The formation of the ATXN1-CIC complex upon polyQ expansion is critical for the onset and severity of SCA1 phenotypes.^4^ Building on this previous work, the researchers hypothesized that directly targeting the ATXN1 gene with CRISPR-Cas9 could reduce ATXN1 protein production, thereby decreasing the toxic gain-of-function interaction with CIC and mitigating disease progression.

They designed two CRISPR-Cas9 strategies to knock down ATXN1 expression to achieve this. The first was a single guide RNA (gRNA) approach targeting the exon-exon junction of exon 8 to induce frameshift mutations leading to nonsense-mediated decay. The second was a dual gRNA approach flanking the CAG repeat region to excise the pathogenic expansion. *In cellulo*, experiments using HEK293 cells expressing human ATXN1 or SCA1 neurons differentiated from patient-derived induced pluripotent stem cells demonstrated that both strategies effectively reduced ATXN1 mRNA and protein levels. Both approaches achieved more than 50% reduction in mRNA and about 40% reduction in protein levels, albeit without mutant allele-specific bias.

To assess the *in vivo* efficacy of these strategies, they next utilized the B05 transgenic mouse model of SCA1, which expresses human ATXN1 with 82 uninterrupted CAG repeats in Purkinje cells. They co-delivered the CRISPR components to the cerebellum using adeno-associated virus (AAV) vectors, administered via intracerebroventricular injections into neonatal mice. Mice treated with the single gRNA approach showed a 54% reduction in ATXN1 mRNA levels in the cerebellum. Behavioral assessments revealed that treated mice exhibited improved motor coordination on the accelerating rotarod test at 20 weeks of age compared to untreated controls. This partial rescue of motor function correlated with the observed reduction level of ATXN1 expression.

**Figure 1:**
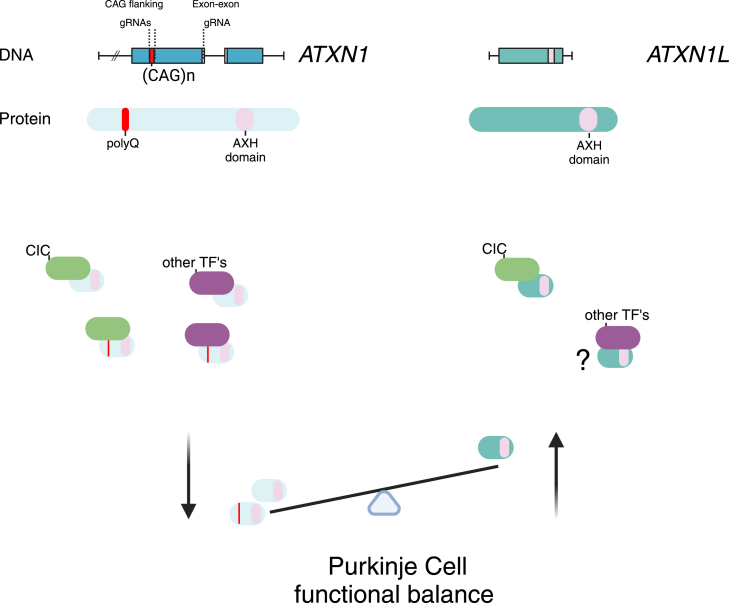
Non-allele-selective ATXN1 knockdown improves Purkinje cell functional balance, leading to improved motor function in a SCA1 mouse model

Integrating these findings with already published literature reveals a complex mechanism involving ATXN1. In SCA1, cerebellar toxicity arises from a toxic gain-of-function mechanism wherein polyQ-ATXN1 abnormally interacts with its native partner CIC and possibly other proteins, including transcription factors (TFs).^5^ ATXN1 removal abolishes such pathological interactions and toxicity without causing detrimental loss-of-function effects in the cerebellum, likely due to compensatory expression of its paralog Ataxin1-like (ATXN1L), which maintains normal CIC levels and essential cellular functions^4^^,^^6^ (see Figure 1). Conversely, in the cortex, loss of ATXN1 function leads to decreased CIC levels, resulting in increased expression of the TFs ETV4 and ETV5. These factors upregulate β-site amyloid precursor protein-cleaving enzyme 1 (BACE1), a key enzyme involved in amyloid-β production implicated in Alzheimer’s disease (AD) pathology.^7^ This upregulation is specific to the adult cerebrum, where ATXN1 levels are higher during adulthood, and ATXN1L may not be sufficiently expressed to compensate for ATXN1 loss, leading to dysregulated transcriptional control. Therefore, while the cerebellum is sensitive to ATXN1’s toxic gain of function but resilient to its loss due to ATXN1L compensation, the cortex can be vulnerable to ATXN1’s loss-of-function effects. These insights highlight that reducing ATXN1 levels will diminish the pathological polyQ-ATXN1-CIC and other possible TF interactions in the cerebellum and could be a fruitful therapeutic strategy for treating ataxia in SCA1. However, it will be necessary to spare other brain regions to maintain critical ATXN1 functions that perhaps ATXN1L cannot compensate for in those regions.

Despite potential risks of off-target effects and immune responses in gene editing therapies, the study demonstrated the high specificity of the gRNAs, finding minimal insertion or deletion (indel) formation at predicted off-target sites (such as ATXN1L) and no significant increase in inflammation markers (GFAP and Iba1) in cerebellar tissues—indicating a favorable safety profile. Nevertheless, using the B05 mouse model, which expresses multiple copies of the human ATXN1 transgene, may affect CRISPR-Cas9 editing efficiency due to the increased number of target sites. Advancing toward clinical application will require validating dose delivery in a knockin SCA1 mouse model, where ATXN1 mRNA levels remain stable with age.

The successful application of CRISPR-Cas9 in SCA1 models opens new avenues for treating not only SCA1 but also other neurological disorders where similar mechanisms are at play. Refining delivery methods could enhance transduction efficiency and specificity, such as blood-brain-barrier-penetrant vectors that activate only in specific cerebellar types. Additionally, transient Cas9 expression via inducible systems or delivery of Cas9 ribonucleoprotein complexes may reduce off-target effects and immune reactions. Comprehensive long-term studies are essential to evaluate the durability of therapeutic effects and monitor for potential late-onset side effects.

This study represents a significant advancement in pursuing effective therapies for SCA1. By demonstrating that CRISPR-Cas9-mediated gene editing can reduce ATXN1 expression and ameliorate disease symptoms in preclinical models, it lays the groundwork for future translational research. As gene editing technologies evolve, there is optimism that integrating CRISPR-Cas9 into clinical practice will eventually lead to durable, disease-modifying treatments for SCA1 and other monogenic neurodegenerative diseases that are currently incurable.

## Acknowledgments

Research in the G.B. lab is supported by the UK Dementia Research Institute, which receives contributions from UK DRI, Ltd. and the UK MRC. Research at TINS is supported by the Romanian Ministry of Research, Innovation and Digitization (no. PNRR-III-C9-2022-I8-66; contract 760114). Graphics were created with BioRender.

## Author contributions

G.B. wrote the manuscript with help from M.M.

## Declaration of interests

G.B. is the founder, director, and CEO of Function RX Ltd.
